# Default mode network connectivity and treatment response in geriatric depression

**DOI:** 10.1002/brb3.2475

**Published:** 2022-03-01

**Authors:** Lisa A. Kilpatrick, Beatrix Krause‐Sorio, Prabha Siddarth, Katherine L. Narr, Helen Lavretsky

**Affiliations:** ^1^ G. Oppenheimer Family Center for Neurobiology of Stress and Resilience Vatche and Tamar Manoukian Division of Digestive Diseases David Geffen School of Medicine at University of California Los Angeles California USA; ^2^ Jane and Terry Semel Institute for Neuroscience and Human Behavior University of California Los Angeles California USA; ^3^ Brain Mapping Center Departments of Neurology and Psychiatry and Biobehavioral Sciences Los Angeles California USA

**Keywords:** default mode network, depression, memory, randomized controlled trial

## Abstract

**Objectives:**

Default mode network (DMN) connectivity is altered in depression. We evaluated the relationship between changes in within‐network DMN connectivity and improvement in depression in a subsample of our parent clinical trial comparing escitalopram/memantine (ESC/MEM) to escitalopram/placebo (ESC/PBO) in older depressed adults (NCT01902004).

**Methods:**

Twenty‐six participants with major depression (age > 60 years) and subjective memory complaints underwent treatment with ESC/MEM (*n* = 13) or ESC/PBO (*n* = 13), and completed baseline and 3‐month follow‐up resting state magnetic resonance imaging scans. Multi‐block partial least squares correlation analysis was used to evaluate the impact of treatment on within‐network DMN connectivity changes and their relationship with symptom improvement at 3 months (controlling for age and sex).

**Results:**

A significant latent variable was identified, reflecting within‐network DMN connectivity changes correlated with symptom improvement (*p* = .01). Specifically, although overall group differences in within‐network DMN connectivity changes failed to reach significance, increased within‐network connectivity of posterior/lateral DMN regions (precuneus, angular gyrus, superior/middle temporal cortex) was more strongly and positively correlated with symptom improvement in the ESC/MEM group (*r* = 0.97, 95% confidence interval: 0.86–0.98) than in the ESC/PBO group (*r* = 0.36, 95% confidence interval: 0.13–0.72).

**Conclusions:**

Increased within‐network connectivity of core DMN nodes was more strongly correlated with depressive symptom improvement with ESC/MEM than with ESC/PBO, supporting an improved engagement of brain circuitry implicated in the amelioration of depressive symptoms with combined ESC/MEM treatment in older adults with depression and subjective memory complaints.

## Abstract 2

We evaluated the relationship between changes in DMN connectivity and improvement in depression in a subsample of our parent clinical trial comparing escitalopram/memantine (ESC/MEM) to escitalopram/placebo (ESC/PBO) in older depressed adults (NCT01902004). A significant latent variable was identified, reflecting DMN connectivity changes related to symptom improvement. Increased connectivity of core DMN nodes was more strongly associated with depressive symptom improvement with ESC/MEM than with ESC/PBO, supporting a neuroplastic effect of memantine addition to escitalopram on brain circuitry that can help reduce depressive symptoms and prevent cognitive decline.

## 1 INTRODUCTION

Late‐life depression (LLD) is common, affecting 10–15% of individuals aged 60 years and older (Ismail et al., 2013), and is associated with cognitive impairment, including memory impairments, which can persist after successful treatment for depression (Diniz et al., 2013; Mitchell & Subramaniam, 2005; Nebes et al., 2003; Singh‐Manoux et al., 2017; Wilkins et al., 2009). Older adults with both mild cognitive impairment and depression are more likely to develop dementia than those with mild cognitive impairment only (Mourao et al., 2016). Additionally, endorsement of subjective memory complaints is associated with increased risk for mild cognitive impairment and dementia. These findings suggest that treatment strategies targeting both depression and memory impairment in its early stages are needed. Memantine (MEM) is a non‐competitive N‐methyl‐D‐aspartate receptor (NMDAR) antagonist that is currently FDA‐approved for the treatment of cognitive symptoms in dementia (McShane et al., 2006). In a double‐blind, randomized placebo‐controlled trial of geriatric major depression with subjective memory complaints (NCT01902004), we previously showed that although escitalopram combined with MEM (ESC/MEM) and ESC combined with placebo (ESC/PBO) both showed improvement in mood, ESC/MEM was more effective than ESC/PBO in improving cognitive outcomes at 12 months (Lavretsky et al., 2020).

The default mode network (DMN) is a large‐scale brain network, mainly comprising the precuneus and posterior cingulate cortex (posterior DMN), medial prefrontal cortex (anterior DMN), and inferior parietal lobe and lateral temporal cortex (lateral DMN), that is involved in a variety of functions, including social cognition, episodic memory, and self‐referential processes (Greicius & Menon, 2004; Gusnard et al., 2001; Iacoboni et al., 2004). DMN alterations are thought to play an important role in the pathophysiology of major depressive disorders (MDD). Although early studies on the DMN in MDD, and specifically LLD, largely reported increased connectivity within the DMN (either between specific nodes in region of interest analyses or between specific nodes and the DMN as a whole in independent component analyses; hitherto referred to as within‐network DMN connectivity) (Andreescu et al., 2013; Hamilton et al., 2015; Kaiser et al., 2015; Li et al., 2013), a more recent large‐scale study found decreased within‐network DMN connectivity as associated with recurrent MDD and antidepressant use (Yan et al., 2019). Furthermore, treatment studies of major depression in both younger and older adults have shown that changes in within‐network DMN connectivity with antidepressant treatment are complex, and can differ depending on treatment response (Andreescu et al., 2013; Karim et al., 2017; Li et al., 2013).

In the present study, we aimed to evaluate the changes in DMN connectivity following 3 months of treatment with ESC/MEM or ESC/PBO, as well as, the relationship between DMN connectivity changes and changes in depressive symptoms, in adults with LLD and subjective memory complaints. We hypothesized that ESC/MEM treatment would show more pronounced changes in within‐network DMN connectivity, associated with greater depressive symptom improvement.

## 2 MATERIALS AND METHODS

### 2.1 Participants

Twenty‐six older adults (age > 60 years) diagnosed with MDD who participated in a parent randomized placebo‐controlled clinical trial (RCT; NCT01902004) of combined escitalopram‐memantine (ESC/MEM) versus escitalopram‐placebo (ESC/PBO) (Lavretsky et al., 2020) and completed resting‐state magnetic resonance imaging (rsMRI) at baseline and 3 months of follow up (total *N* = 26, ESC/MEM, *N* = 13; ESC/PBO, *N* = 13) were included. The placebo comprised a capsule containing an inert substance. Eligibility criteria for the RCT were as follows: 1) At least 60 years of age; 2) a diagnosis of MDD according to the Diagnostic and Statistical Manual (DSM‐5) diagnostic criteria (APA Association, 2013) and a score of at least 16 on Hamilton Rating Scale for Depression (Hamilton, 1960); 3) an absence of dementia, determined by neurological evaluations, neuropsychological evaluations, laboratory tests (see Lavretsky et al., 2020 for details) and a score > 23 on the Mini‐Mental State Examination (Folstein et al., 1975); and 4) subjectively reported memory impairment. Exclusion criteria were as follows: 1) A prior history of psychiatric disorders, such as, substance abuse disorder, suicidal behavior, or suicide attempts in the past year; 2) acute, severe, or recent medical illness; and 3) a history of allergies or intolerance to either escitalopram or memantine. Additionally, participants who consented to the neuroimaging component could not have any MRI‐incompatible implants or other imaging contra‐indications. None of the participants took cognitive enhancers at study entry.

The study was approved by the UCLA Institutional Review Board, and written informed consent was obtained from all participants.

### 2.2 Study design

Details regarding the study protocol have been previously described (Lavretsky et al., 2020). Participants remained free from psychotropic medication for at least two weeks before baseline assessments (four weeks if they had previously taken fluoxetine). The daily dose of escitalopram was 10 mg for the first four weeks of administration, and was increased to 20 mg after four weeks if the Clinical Global Impression Scale (Guy, 1976) score was at least 3. The daily dose of memantine or matched placebo was gradually increased from 5 to 20 mg over the course of four weeks. Daily study drug doses were adjusted to a minimum of 5 mg for memantine and 10 mg of escitalopram, based on tolerability.

Baseline and 3‐month follow‐up assessments included the Montgomery‐Asberg Depression Rating Scale (MADRS) (Montgomery & Asberg, 1979) as a measure of depressive symptom severity, which has been shown to be sensitive to combined ESC/MEM treatment (Lavretsky et al., 2020). Symptom improvement scores were calculated as follows: (−1 X (3‐month score—baseline score)).

### 2.3 Neuroimaging

MRI‐eligible participants underwent scanning at baseline and the 3‐month follow up. A high‐resolution T1‐weighted MRI image and resting state BOLD images were collected using either a 3T Siemens TIM Trio (ESC/MEM: *n* = 5; ESC/PBO; *n* = 7) or Prisma‐fit system (Siemens, Erlangen, Germany; ESC/MEM: *n* = 8; ESC/PBO; *n* = 6) with a 32‐channel head coil. For each participant, baseline and follow‐up scans were collected on the same MRI system. The multi‐echo MPRAGE scan was collected using the following parameters, matched across scanners: Isotropic 1‐mm^3^ (Trio) or 0.8‐mm^3^ (Prisma) voxels; 176 (Trio) or 208 (Prisma) slices; TR 2150 (Trio) or 2400 (Prisma) ms; TE, 1.74, 3.6, 5.46, and 7.32 (Trio) or 2.24 (Prisma) ms; TI, 1260 (Trio) or 1060 (Prisma); FOV, 256 mm; matrix size, 256 × 256 (Trio) or 256 × 240 (Prisma) mm; and flip angle, 7 (Trio) or 8 (Prisma) degrees. The 5.7‐min (Trio)/7.3‐min (Prisma) resting state scan (eyes closed) was collected using the following parameters, matched across scanners: isotropic 1.8‐mm^3^ voxels; 78 slices; TR 1240 (Trio) or 1595 (Prisma) ms; TE, 38.2 (Trio) or 49.8 (Prisma) ms; FOV, 212 (Trio) or 213 (Prisma) mm; matrix size, 212 × 212 (Trio) or 213 × 213 (Prisma) mm; and flip angle, 65°.

**FIGURE 1:**
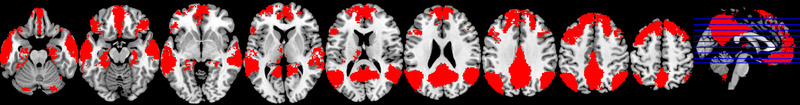
The DMN mask used in the partial least squares correlation analysis, identified by group independent component analysis

Image quality was assessed using MRIQC (Esteban et al., 2017). Imaging data were pre‐processed using fMRIPREP version 1.4.1rc1 (RRID:SCR_016216) (Esteban et al., 2019). Resting state data were motion corrected, followed by co‐registration to the corresponding T1‐weighted structural scan and normalization to MNI space. The data was smoothed with a kernel width of 6 mm, and independent component analysis‐based automatic removal of motion artifacts (ICA‐AROMA) was used to non‐aggressively denoise the data (Pruim et al., 2015). Preprocessing results were visually inspected using the summary reports provided by fMRIPREP (Esteban et al., 2019). Group‐ICA was implemented with the FSL multivariate exploratory linear optimized decomposition into independent components (MELODIC) algorithm with a dimensionality of 25 (http://www.fmrib.ox.ac.uk/fsl/melodic/index.html) (Beckmann & Smith, 2005). A single DMN component, comprising both anterior and posterior DMN key nodes (precuneus and medial prefrontal cortices), was identified (Figure 1). Dual regression was performed to create individual‐participant DMN parameter estimate maps (Nickerson et al., 2017). For each participant, the 3‐month change in within‐network DMN connectivity was calculated by subtracting the baseline DMN map from the 3‐month DMN map.

### 2.4 Statistical analysis

Group differences in clinical characteristics were evaluated using the non‐parametric Kruskal‐Wallis test for continuous variables and the Fisher's exact test for categorical variables. Group differences in baseline within‐network DMN connectivity were evaluated in an unpaired t‐test using randomize in FSL (Winkler et al., 2014). Partial least squares correlation (PLSC) analysis was applied to simultaneously evaluate group differences in DMN connectivity changes and the relationships between DMN connectivity changes and symptom improvement at 3 months. PLSC is a multivariate statistical technique that identifies relationships between patterns in two or more blocks of variables (e.g., neuroimaging data, behavioral/group data) and is considered more sensitive than traditional univariate approaches (Krishnan et al., 2011; McIntosh & Lobaugh, 2004; McIntosh et al., 1996). In PLSC, latent variables, comprising a set of spatial maps with associated weightings for each group/behavioral measure, are produced. PLSC was implemented using freely available code (http://www.rotman‐baycrest.on.ca/pls) (McIntosh et al., 1996)^.^ Sex‐ and age‐adjusted individual‐participant DMN maps and the 3‐month improvement in MADRS scores were submitted as input to the PLSC analysis, and to limit the analysis to regions within the DMN, a mask of the brain regions with positive weights on the group DMN map produced by the ICA analysis was applied. Significance of each latent variable was assessed using permutation testing (5000 permutations), and voxel reliability was assessed using bootstrap estimation (5000 samples) (McIntosh et al., 1996). Clusters with a peak voxel bootstrap standard error ratio (BSR) exceeding ±3.3 (*p* < .001) and an extent of at least 125 voxels are reported. For all other measures, *p* < .05 was considered statistically significant.

## 3 RESULTS

### 3.1 Baseline data

Baseline demographics and clinical data are summarized in Table 1. There were no significant baseline differences between groups in demographics (age, sex, education, and ethnicity) and clinical data (Mini‐Mental State Examination, diagnosis of MCI, depressive and cognitive measures).

**TABLE 1:** Baseline characteristics according to group

	**ESC/MEM (*n* = 13)**		**ESC/PBO (*n* = 13)**		**Statistics**
**Variable**	N	%	N	%	Fisher's exact test
Sex: Female	7	53.85	8	61.54	*p* > .99
Race/ethnicity					*p* > .99
Caucasian	10	76.92	11	84.62	
Hispanic	3	23.08	2	15.38	
Diagnosis of MCI	2	15.38	2	15.38	*p* > .99

	M	SD	M	SD	Kruskal‐Wallis test
Age (years)	70.38	7.56	69.54	7.46	χ2(1) = 0.04, *p *= .84
Education (years)	15.77	2.28	15.93	2.22	χ2(1) = 0.14, *p* = .71
MMSE	28.92	.95	28.08	1.66	χ2(1) = 1.83, *p* = .18
MADRS	16.62	2.43	15.54	2.82	χ2(1) = 1.75, *p *= .19

Abbreviations: ESC, Escitalopram; MEM, Memantine; PBO, Placebo; MCI, Mild Cognitive Impairment; MMSE, Mini‐Mental State Examination; MADRS, Montgomery–Åsberg Depression Rating Scale.

### 3.2 Symptom improvement

Although the post‐treatment improvement in MADRS scores relative to the pretreatment score was greater in the ESC/MEM group (mean ± standard deviation: −10.23 ± 6.17) than in the ESC/PBO group (−6.62 ± 3.97), the group difference failed to reach significance (χ^2^(1) = 3.45; *p* = .06).

### 3.3 Within‐network default mode network connectivity

**FIGURE 2:**
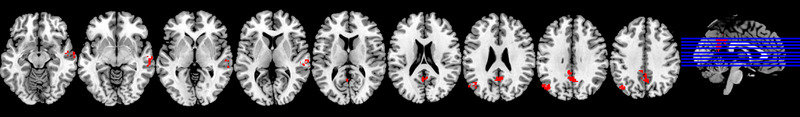
Regions showing a stronger relationship between increased within‐network DMN connectivity following treatment and symptom improvement in the ESC/MEM group than in the ESC/PBO group. DMN, default mode network; ESC escitalopram; MEM, memantine; PBO, placebo

There were no significant group differences in baseline within‐network DMN connectivity. However, the PLSC analysis identified a single significant latent variable (accounting for 24.9% of the cross‐block variance, *p* = .01), reflecting a non‐significant group difference in within‐network DMN connectivity changes and a significant group difference in the relationship between within‐network DMN connectivity changes and symptom improvement. Specifically, the PLSC identified a set of regions that tended to show larger within‐network DMN connectivity changes in the ESC/MEM group than in the ESC/PBO group; however, the group difference failed to reach significance. In these same regions, increased within‐network connectivity was significantly more positively correlated symptom improvement in the ESC/MEM group (*r* = 0.97, 95% confidence interval: 0.86–0.98) than in the ESC/PBO group (*r* = 0.36, 95% confidence interval: 0.13 to 0.72). Additionally, in this set of regions, the group difference in relationship between within‐network DMN connectivity changes and symptom improvement remained significant when controlling for scanner type (partial correlation with bootstrapping; ESC/MEM group: *r* = 0.97, 95% confidence interval: 0.79–0.99; ESC/PBO group: *r* = 0.13; 95% confidence interval: −0.38 to 0.74). Regions reliably contributing to this pattern included several core DMN regions (precuneus, angular gyrus, and middle/superior temporal cortex) (Table 2, Figure 2). In the PLSC analysis, no areas with negative weights on the identified latent variable (indicating decreased DMN connectivity as more positively correlated symptom improvement in the ESC/MEM group than in the ESC/PBO group) survived the bootstrap estimation of reliability.

**TABLE 2:** Regions reliably showing a stronger correlation between within‐network connectivity increases and symptom improvement in the ESC/MEM group than in the ESC/PBO group

**Region**	**MNI cords**	**BSR**	**Cluster size**
R middle temporal gyrus	56 −2 −16	10.5	126
L angular gyrus	−50 −70 32	10.0	170
Bilat precuneus	2 −58 38	10.0	412
R superior temporal gyrus	62 −24 −4	9.0	137

*Note*: All *p* < .001.

Abbreviations: L, left; R, right; Bilat, bilateral; BSR, bootstrap ratio; MNI coords, Montreal Neurological Institute coordinates; DMN, default mode network; ESC, escitalopram; MEM, memantine; PBO, placebo.

## 4 DISCUSSION

Escitalopram is a selective serotonin reuptake inhibitor used for the treatment of geriatric depression (Cipriani et al., 2009; Wu et al., 2008). Memantine is a low‐to‐moderate affinity noncompetitive NMDA receptor antagonist currently used to slow cognitive decline in patients with Alzheimer disease (Gellis et al., 2009). Our parent RCT showed that combined ESC/MEM treatment was more effective than ESC/PBO treatment in improving depressive symptoms (in terms of MADRS scores) at 6 months and cognitive function at 12 months in patients with LLD and subjective memory complaints, and without dementia (Lavretsky et al., 2020). Thus, ESC/MEM treatment may be effective in targeting both depression and memory impairment in older adults. However, little is known regarding the neurocircuitry underlying this additional benefit. In the present study, we evaluated the 3‐month change in within‐network DMN connectivity following treatment with ESC/MEM or ESC/PBO for LLD with subjective memory impairments, as well as, the relationship between DMN connectivity change and symptom improvement. We found that although overall differences in the change in within‐network DMN connectivity between the two groups failed to reach significance, the groups differed in the strength of the relationship between within‐network DMN connectivity increases and depressive symptom improvement as assessed by the MADRS. Increased within‐network connectivity of core posterior and lateral DMN nodes was more strongly positively correlated with symptom improvement in patients treated with ESC/MEM than in those treated with ESC/PBO.

A previous treatment study evaluating within‐network DMN connectivity changes showed that changes in within‐network DMN connectivity can occur shortly after medication exposure, within 1 week for venlafaxine, depending on whether remission is subsequently achieved (Karim et al., 2017). This result suggests that early changes in functional connectivity can predict better longer‐term clinical outcomes. Both groups in the present study showed similar symptom improvement over the 3‐month study period likely due to treatment with escitalopram, as well as, a similar change in within‐network DMN connectivity; however, the relationship between the increase in within‐network connectivity of key DMN regions and symptom improvement was stronger with the additional administration of memantine. This difference in the strength of the relationship between brain and behavioral changes suggest more robust functional reorganization relevant to depression with ESC/MEM than with ESC/PBO, possibly due to greater transcriptomic gene expression changes in the neuroplastic pathways with memantine treatment based on our previous report (Grzenda et al., 2020). As early changes in functional connectivity may predict better longer‐term clinical outcomes, we speculate that this more robust functional reorganization underlies the better long‐term outcomes with ESC/MEM than with ESC/PBO observed in the larger parent study and transcriptome analyses (Grzenda et al., 2020; Lavretsky et al., 2020).

Numerous studies have investigated the direction and nature of pathological DMN connectivity in depression, as well as, the impact of treatment on DMN connectivity, with conflicting results. Early neuroimaging studies on depression and dysthymia reported mainly increased DMN connectivity (Andreescu et al., 2013; Hamilton et al., 2015; Kaiser et al., 2015; Li et al., 2013; Posner et al., 2013), suggesting that DMN connectivity reduction may be a treatment target. However, a recent large‐scale study showed more complex results, with increased orbitofrontal and decreased posterior DMN connectivity in MDD (Yan et al., 2019). Although a previous MDD treatment study reported decreased within‐network connectivity for posterior DMN regions following antidepressant treatment, a prior LLD treatment study reported increased DMN connectivity for posterior DMN regions (precuneus) and decreased DMN connectivity for frontal regions (superior frontal and precentral gyri) following antidepressant treatment (Andreescu et al., 2013). Another LLD study reported a greater increase in DMN connectivity in lateral DMN regions (middle temporal gyrus) and a greater DMN connectivity decrease in DMN connectivity in frontal regions (inferior frontal gyrus) following venlafaxine treatment in remitters than in non‐remitters (Andreescu et al., 2013; Karim et al., 2017). Consistent with these latter studies, the present study found that greater symptom improvement with standard antidepressant treatment using escitalopram correlated with increased within‐network connectivity of the posterior and lateral DMN regions (precuneus, angular gyrus, superior/middle temporal gyrus), and this relationship was strengthened with the addition of memantine. Overall, recent studies suggest increased within‐network connectivity of posterior/lateral DMN nodes as a target in ameliorating depressive symptoms.

The present study has several limitations to acknowledge. Many patients who enrolled in our RCT were unable to participate in the neuroimaging component due to existing contraindications such as, metal implants, common among older adults, which resulted in a small sample size. Additionally, two different scanners were used. However, ICA methods, including ICA‐AROMA preprocessing, generally perform well, showing good spatial reproducibility and similar test‐retest reliability across scanners (Marchitelli et al., 2016; Parkes et al., 2018). Furthermore, the study design did not include an untreated control group. Finally, although there is consensus regarding the key nodes of the DMN, various methods can be used to define the DMN (e.g., ICA, precuneus seed correlation analysis, selection of ROIs based on the literature). This variability may account for some of the differences between studies regarding pathological within‐network DMN connectivity and antidepressant treatment effects on within‐network DMN connectivity, rendering it more difficult to reach a consensus regarding DMN‐related treatment targets.

## 5 CONCLUSIONS

Increased within‐network connectivity of posterior and lateral nodes of the DMN under 3 months of escitalopram treatment correlated with greater improvement in depression severity. However, this relationship was increased with the addition of memantine compared to placebo, thus supporting our prior observations of a greater neuroplastic potential with the adjunct use of memantine for the treatment of geriatric depression. The present study also further supports the importance of the DMN in depression and adds to the limited literature on its role in treatment‐related effects in LLD. In addition, the present study supports an improved engagement of brain circuitry implicated in the amelioration of depressive symptoms with combined ESC/MEM treatment in adults with LLD and subjective memory complaints.

## CONFLICT OF INTEREST

The authors declare no conflicts of interest.

### PEER REVIEW

The peer review history for this article is available at https://publons.com/publon/10.1002/brb3.2475

## Data Availability

The data that support the findings of this study are available on request from the corresponding author. The data are not publicly available due to privacy or ethical restrictions.
